# Post-processing steps improve generalisability and robustness of an MRI-based radiogenomic model for human papillomavirus status prediction in oropharyngeal cancer

**DOI:** 10.1007/s00330-025-11709-8

**Published:** 2025-06-06

**Authors:** Milad Ahmadian, Zuhir Bodalal, Paula Bos, Roland M. Martens, Georgios Agrotis, Hedda J. van der Hulst, Conchita Vens, Luc Karssemakers, Abrahim Al-Mamgani, Pim de Graaf, Bas Jasperse, Ruud H. Brakenhoff, C. René Leemans, Regina G. H. Beets-Tan, Jonas A. Castelijns, Michiel W. M. van den Brekel

**Affiliations:** 1 Department of Head and Neck Oncology and Surgery, The Netherlands Cancer Institute/Antoni van Leeuwenhoek Hospital, Amsterdam, The Netherlands; 2 Department of Radiology, The Netherlands Cancer Institute/Antoni van Leeuwenhoek Hospital, Amsterdam, The Netherlands; 3 Amsterdam Center for Language and Communication, University of Amsterdam, Amsterdam, The Netherlands; 4 GROW School for Oncology and Developmental Biology, Maastricht University, Maastricht, The Netherlands; 5 Department of Radiation Oncology, The Netherlands Cancer Institute/Antoni van Leeuwenhoek Hospital, Amsterdam, The Netherlands; 6 Department of Radiology and Nuclear Medicine, Amsterdam UMC, Location Vrije Universiteit Amsterdam, Amsterdam, The Netherlands; 7 Department of Radiology, University Hospital of Larissa, Thessaly, Greece; 8 School of Cancer Science, University of Glasgow, Glasgow, UK; 9 Department of Otolaryngology-Head and Neck Surgery, Amsterdam UMC, Location Vrije Universiteit Amsterdam, Amsterdam, The Netherlands; 10 Cancer Center Amsterdam, Imaging and Biomarkers, Amsterdam, The Netherlands; 11 Faculty of Health Sciences, University of Southern Denmark, Odense, Denmark

**Keywords:** Radiomics, Imaging genomics, Machine learning, Magnetic resonance imaging, Human papillomavirus

## Abstract

**Objectives:**

To assess the impact of image post-processing steps on the generalisability of MRI-based radiogenomic models. Using a human papillomavirus (HPV) status in oropharyngeal squamous cell carcinoma (OPSCC) prediction model, this study examines the potential of different post-processing strategies to increase its generalisability across data from different centres and image acquisition protocols.

**Materials and methods:**

Contrast-enhanced T1-weighted MR images of OPSCC patients of two cohorts from different centres, with confirmed HPV status, were manually segmented. After radiomic feature extraction, the HPV prediction model trained on a training set with 91 patients was subsequently tested on two independent cohorts: a test set with 62 patients and an externally derived cohort of 157 patients. The data processing options included: data harmonisation, a process to ensure consistency in data from different centres; exclusion of unstable features across different segmentations and scan protocols; and removal of highly correlated features to reduce redundancy.

**Results:**

The predictive model, trained without post-processing, showed high performance on the test set, with an AUC of 0.79 (95% CI: 0.66–0.90, *p* < 0.001). However, when tested on the external data, the model performed less well, resulting in an AUC of 0.52 (95% CI: 0.45–0.58, *p* = 0.334). The model’s generalisability substantially improved after performing post-processing steps. The AUC for the test set reached 0.76 (95% CI: 0.63–0.87, *p* < 0.001), while for the external cohort, the predictive model achieved an AUC of 0.73 (95% CI: 0.64–0.81, *p* < 0.001).

**Conclusions:**

When applied before model development, post-processing steps can enhance the robustness and generalisability of predictive radiogenomics models.

**Key Points:**

***Question*** *How do post-processing steps impact the generalisability of MRI-based radiogenomic prediction models?*

***Findings*** *Applying post-processing steps, i.e., data harmonisation, identification of stable radiomic features, and removal of correlated features, before model development can improve model robustness and generalisability.*

***Clinical relevance*** *Post-processing steps in MRI radiogenomic model generation lead to reliable non-invasive diagnostic tools for personalised cancer treatment strategies.*

**Graphical Abstract:**

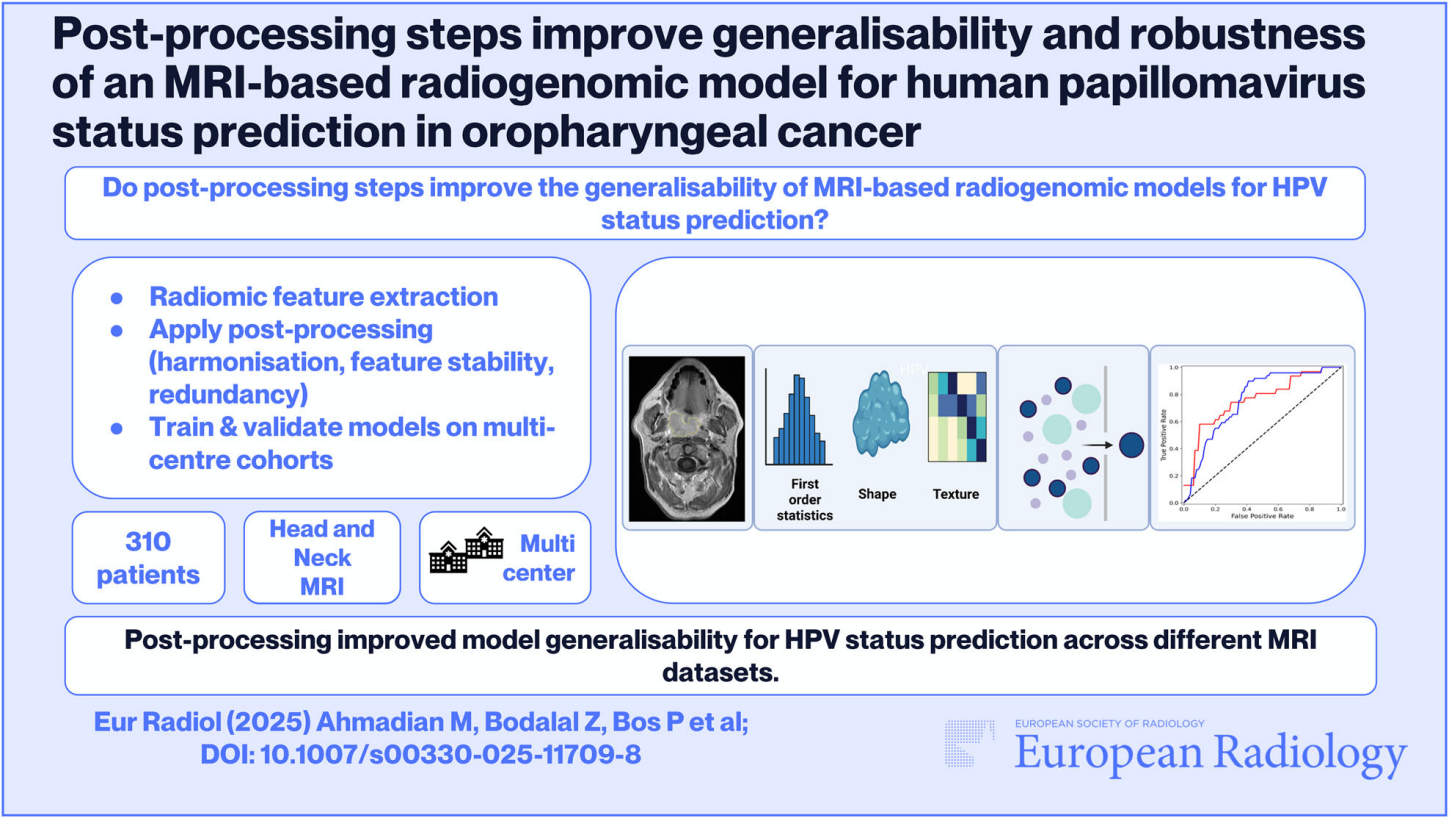

## Introduction

Radiomics has become a valuable technique for extracting quantitative information from medical images to characterise tumour phenotypes [1, 2]. The extracted radiomic features have also been linked to the underlying biology, a field referred to as radiogenomics [3, 4]. Numerous research studies have developed radiogenomic models to predict human papillomavirus (HPV) status (an established prognostic and predictive biomarker) in oropharyngeal squamous cell carcinoma (OPSCC) using various imaging modalities, including computed tomography (CT) [5, 6], positron emission tomography (PET)/CT [7, 8], and magnetic resonance imaging (MRI) [9, 10]. These investigations have yielded promising outcomes, demonstrating that radiomics-based approaches hold significant potential as a non-invasive method for determining HPV status in oropharyngeal cancer patients. Nevertheless, for radiomics-based HPV prediction models to be applicable in clinical practice, they must be both robust and generalisable to levels similar or superior to standard HPV diagnostic by molecular assays, even in the presence of variations in image scan protocols, acquisition parameters, and tumour segmentations, which typically differ across multicentre analysis [11, 12].

The validation of radiogenomics models across multicentre cohorts has been explored in some studies, but significant challenges remain in ensuring broad applicability and enhancing the generalisability of these predictive techniques, especially in the context of MR imaging [13, 14]. This issue arises from the high variability in MRI techniques, including differences in image acquisition and reconstruction parameters, which can introduce limitations in developing radiogenomics models when dealing with multicentre cohorts [15]. Differences in MRI field strength, such as 1.5-Tesla and 3.0-Tesla, and MRI manufacturers and scan parameters, led to bias in the extracted radiomic features, negatively impacting the generalisability of previously established predictive parameters and models [16–18]. Furthermore, inter- and intra-observer variations in MRI scans result in discrepancies, such as in tumour volume segmentations, especially in head and neck cancer [19, 20]. The implementation of post-processing techniques on the extracted radiomic features may be crucial to address these limitations and enhance the performance of the models across different centres. By removing unstable and correlated radiomic features, such data post-processing techniques would be best performed before modelling to identify features to prevent complicated issues in radiogenomic model generation, such as overfitting and low external performance.

In this study, we aimed to investigate how post-processing strategies applied to radiomic features can enhance the robustness and generalisability of predictive MR-based radiogenomics models. To this end, we used different patient cohorts for training and performance evaluation purposes that comprised patients from two centres, the Netherlands Cancer Institute (NKI) and Amsterdam UMC (AUMC). First, we used the previously published radiomics-based HPV prediction model by Bos et al [21] to train part of the NKI cohort (NKI-training set) and test its performance on an independent NKI-test set, therefore on images from the same centre, and compared those achieved in an imaging dataset from a different centre (AUMC). Next, to address the challenges posed by the differences in MR imaging approaches and hardware, we assessed the impact of various post-processing steps to improve the model robustness and generalisability.

## Materials and methods

### Data collection

In this study, we retrospectively studied baseline MRI scans of two patient cohorts with histologically confirmed primary oropharyngeal squamous cell carcinoma (OPSCC) and known HPV status (positive versus negative), determined as reported below. The first cohort included 153 patients with a pretreatment MRI prior to (chemo-)radiotherapy at the NKI. The second cohort consisted of pretreatment MRI scans from 157 patients treated at the AUMC. Both cohorts were extensively described by Bos et al [22].

HPV status was determined at the NKI using a combined analysis of p16 and p53 immunohistochemistry on OPSCC biopsy material. OPSCC HPV status of AUMC-treated patients was assessed by p16 immunostaining, followed by DNA-PCR in cases exhibiting positive p16 immunoreactivity. The Institutional Review Board (IRB) of the NKI (IRBd19-240) approved the use of the data for the analysis in this study.

### Image acquisition and tumour segmentation

For NKI cohort patients, post-contrast 3D T1-weighted image acquisition was performed using the Achieva scanner from Philips Medical Systems, accounting for 153 scans. In the NKI imaging data, 74 MR scans (48%) were acquired via a magnetic field strength of 1.5 Tesla, while 79 scans (52%) were conducted at 3.0 Tesla. For the AUMC cohort, contrast-enhanced 2D T1-weighted MR images were acquired using a variety of scanners. The Integrity system from Philips Medical Systems was used for 73 scans (46%), the Signa HDxt system from GE Medical Systems accounted for 54 scans (34%), and other devices were used for 31 scans (20%). In the AUMC patient cohort, 80 MR images (51%) utilised a magnetic field of 1.5 Tesla, and 77 scans (49%) were carried out at 3.0 Tesla. Comprehensive MR-image acquisition parameters for both centres are provided in Supplementary Material S1.

In the NKI cohort, tumour segmentation was performed in a two-step process, where initial segmentations were done by P.B. (a technical PhD researcher) and subsequently reviewed and refined by B.J. (an expert radiologist with over 7 years of experience in head and neck tumour delineation). The segmentation process used 3D Slicer (version 4.8.0) on every axial slice of the 3D MR images, with additional MRI sequences available to aid contouring.

Within the AUMC cohort, primary tumour delineation was conducted independently by two experienced head and neck radiologists, J.C. (with over 35 years of MRI-diagnostic head and neck tumour experience) and P.d.G. (with over 13 years of MRI-diagnostic head and neck tumour experience). These radiologists manually segmented the tumours using the VELOCITY software. Following their independent delineations, divergences in tumour segmentation were resolved through a consensus meeting, resulting in a final consensus segmentation that was used in the study. Radiologists used other available pretreatment scans to assist in the manual delineation process.

**Fig. 1:**
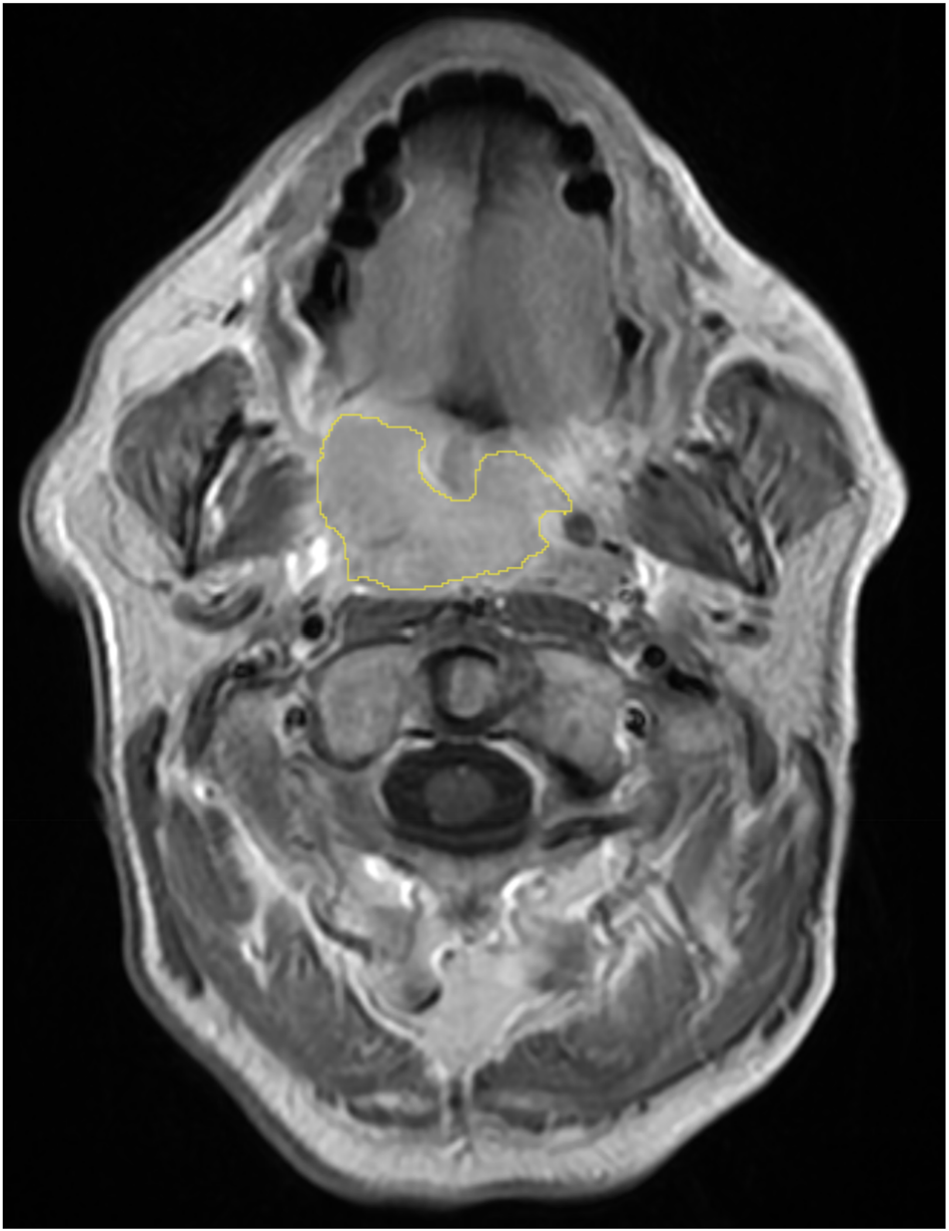
Delineation of primary oropharyngeal carcinoma on contrast-enhanced T1-weighted MRI. This figure demonstrates the segmentation strategy for viable tumour tissue (yellow ROI). Peritumoural inflammation was found with higher intensity surrounding the tumour tissue. Additionally, a necrotic mass was seen with low intensity anterior to the tumour tissue. Both peritumoural inflammation and necrotic mass were excluded by segmentation from viable tumour tissue

Although the process of primary tumour segmentation was separately conducted at both centres, we ensured that consistent segmentation criteria were applied to both cohorts. Specifically, segmentation focused on delineating the viable tumour regions while excluding artefacts, peritumoural inflammation, necrotic and cystic areas. Figure 1 illustrates the segmentation of primary tumours performed on contrast-enhanced T1-weighted MRI scans for quantitative data extraction.

### Radiomic feature extraction

Pyradiomics (v3.0.1a1) was used to extract radiomic features on each segmented primary lesion of both the NKI and AUMC cohort [23]. Before feature extraction, MR images were adjusted to reduce differences in image brightness and mitigate intensity variations across different patients. Notably, normalisation was performed using the voxel intensity values of the entire image, not just within the region of interest. To further preprocess the images, MR scans were resized to have uniform, 1 mm^3^ isotropic voxels using a smoothing technique called B-spline interpolation, performed after tumour segmentation. Then, the images were divided into fixed bins with a width of 5 to measure texture. To align our efforts to the methodology described by Bos et al [21], this was done without performing optimisation. A total of 1184 radiomic features were extracted, including texture, shape, Laplacian of Gaussian filter, wavelet transform, and intensity features. The radiomic features were subjected to standardisation to achieve zero mean and unit variance.

### Study design

**Fig. 2:**
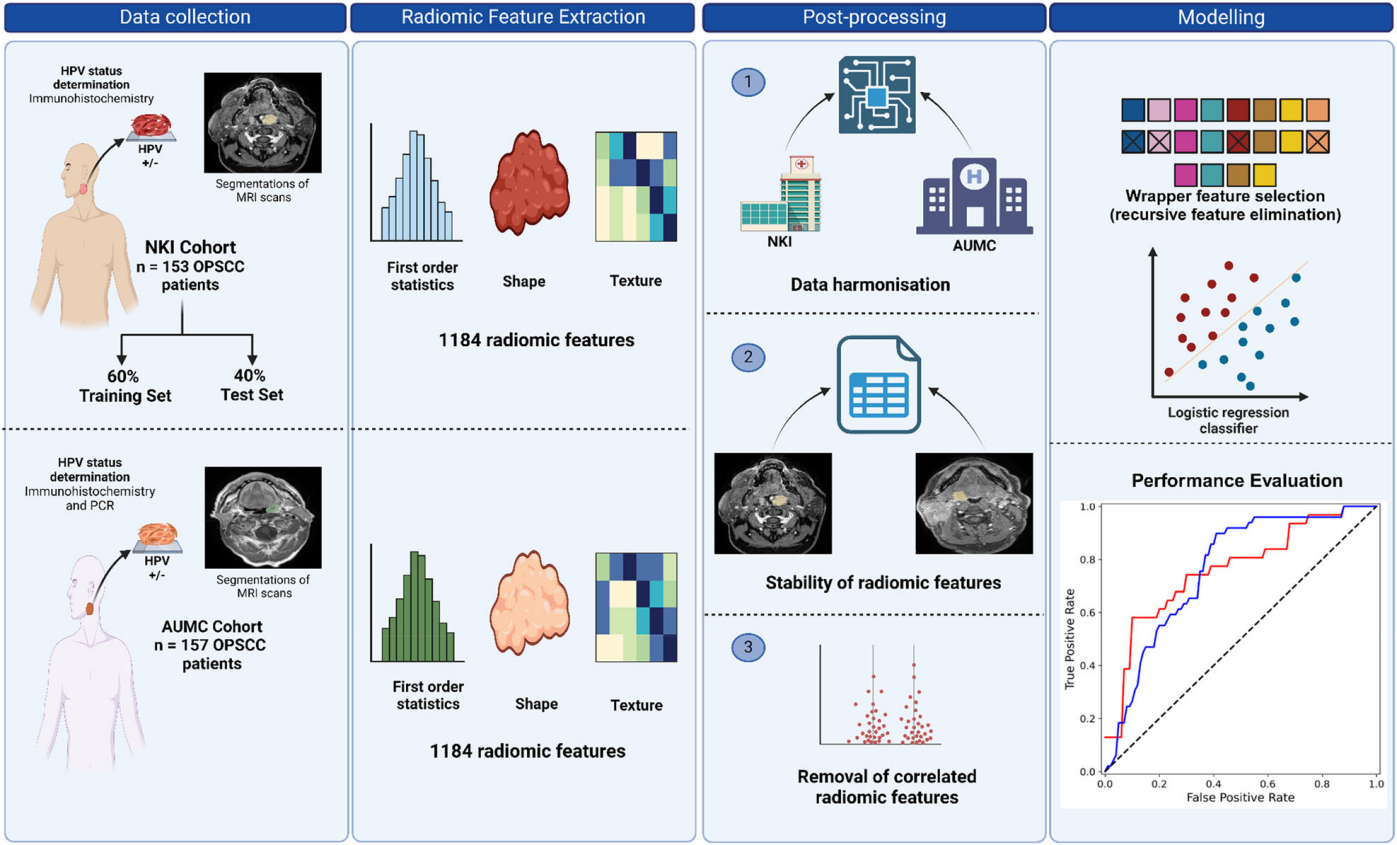
General study design. This study analysed MRI images from two NKI and AUMC oropharyngeal squamous cell carcinoma (OPSCC) cohorts with known HPV status. We first acquired and segmented MR images. Subsequently, radiomic features from both cohorts were extracted. We then performed several post-processing steps, including (1) data harmonisation, (2) identification of stable radiomic features across various segmentations and scan protocols, and (3) the removal of highly correlated radiomic features. The final phase involved modelling and a comprehensive evaluation, focusing on each post-processing step’s impact on the predictive model’s robustness and generalisability

For the purposes of this study, we used the NKI cohort (*n* = 153 patients) for training and internal test, and the external AUMC cohort (*n* = 157 patients) to validate the performance of the model on data from a different centre. The random split of the NKI data between training set (60%, *n* = 91) and test set (40%, *n* = 62) exactly matched Bos et al [21]. Our primary endpoint was to assess how various post-processing steps (as well as harmonisation of external data) might impact the generalisability of our radiogenomics pipeline. Different post-processing strategies to minimise the impact of centre-specific MR imaging protocols and hardware were applied, and models were subsequently trained using the NKI-training set. The trained models were then applied to predict HPV status of the patients in the NKI-test set and AUMC cohort (see Fig. 2). We evaluated the impact of specific post-processing steps on the generalisability and robustness of the HPV radiogenomic model through the following four scenarios:

A) In the first scenario, for comparison and reference purposes, an HPV prediction model was trained on the full number of features (*n* = 1184) derived from the samples in the NKI-training set, without any post-processing steps. The model’s performance was then assessed on the NKI-test set. Subsequently, this model was applied directly to the AUMC cohort.
B) In the second scenario, data variations across different centres were corrected by removing batch effects. To accomplish this, we implemented ComBat harmonisation as described by Johnson [24] to harmonise the radiomic features extracted from the AUMC cohort towards the feature domain of the NKI cohort. ComBat harmonisation uses statistical techniques based on Bayesian estimates and location scaling to adjust the mean and variance of the radiomic features. Only radiomic features were used in the Combat method, and no additional covariates were included. This process aligns the extracted radiomic features from one cohort to the distribution and variability of another cohort, making the data more comparable (implemented using the neuroComBat python package as described by Fortin et al [25]).
C) Third, we aimed to minimise the impact of scanner/protocol level differences and intra-reader variability by identifying features mostly affected by these processes. Within the NKI cohort, the image acquisition utilised two distinct scan protocols, and both expert and non-expert observers performed image segmentation. To mitigate the impact of intra-reader variability, we calculated the intraclass correlation coefficient (ICC) between the radiomic features extracted from both observers. An ICC threshold of > 0.75 was chosen in accordance with established guidelines [26, 27]. Additionally, to address differences arising from varying MRI-scanner and scan protocols, the Mann–Whitney U test was used to assess the association of each radiomic feature with the MR-image acquisition protocol [21, 28, 29]. Features with *p* < 0.05 were considered to have a strong association with the acquisition protocol, thus potentially impacting generalisability and were therefore excluded for further analysis.
D) The last scenario applied the Pearson correlation coefficient on the NKI cohort to remove redundant and highly correlated (coefficient > 0.9) radiomic features. This process helped to minimise multicollinearity among radiomic features and robust radiomic analyses. The resulting features were thus deemed more suitable and appropriate for further radiomic investigations.

Bos et al [21] trained their HPV prediction model by performing the steps as described in both scenarios C and D (dropping unstable and correlated features). In this work, we will also reproduce that specific model and assess generalisability using the external AUMC cohort.

### Machine learning

Across all scenarios, we used a wrapper feature selection method, recursive feature elimination (RFE), before fitting the model to remove irrelevant features using a sequential backward technique. This approach involved iteratively removing radiomic features with the lowest feature importance value as described by Kohavi et al [30]. A logistic regression classifier was used to model the training data. A Bayesian optimisation approach was utilised during the training step to find the optimal model’s hyperparameters through 1000 iterations of 4-fold cross-validation on the NKI-training set. Within this process, we optimised the number of selected features and the regularisation parameter (*λ* = 0.005–200). The obtained optimal hyperparameters were employed to train the model and to evaluate its predictive performance on the NKI-test set and the AUMC cohort. The optimisation procedure was applied using the open-source hyperopt Python package [31].

### Statistical analysis

The model’s predictive performance was evaluated using the area under the receiver operating characteristic curve (AUC), accuracy, specificity, sensitivity, the positive predictive value (PPV), and the negative predictive value (NPV). Bootstrapping with 1000 iterations was conducted for all evaluation metrics to calculate the median and 95% confidence intervals (CI) of all reported metrics. The differences between clinical characteristics of different cohorts were calculated using an independent Student’s *t*-test and Chi-squared test. Principal Component Analysis (PCA) was used to visually demonstrate the variation of radiomic features extracted from both cohorts under different post-processing steps.

All analyses were conducted using Python 3.8.16 (numpy 1.19.3, scikit-learn 0.22.2, pandas 1.1.5, matplotlib 3.3.0, seaborn 0.12.2, hyperpipe, hyperopt 0.2.5, tqdm 4.65.0, scipy 1.6.0, statsmodels 0.10.2, pyradiomics v3.0.1a1, neuroComBat 0.2.12).

## Results

### Patient cohorts description

To emulate the difficulties in generalisability of predictive radiogenomic models across different centres and imaging protocols and capacities, we used oropharyngeal cancer datasets of baseline MRI images and matched HPV status from two centres in this study. In the first centre, 153 patients were treated and imaged at the NKI. The second centre’s dataset was derived from 157 patients treated and imaged at the AUMC. Detailed patient characteristics can be found in Table 1. Most demographic characteristics exhibited a similar distribution in both cohorts (NKI: 63% males, AUMC: 71% males, *p* = 0.292). There was no statistically significant difference in the median age between the two groups (NKI: 61 years (IQR: 0.56–0.66), AUMC: 61 years (IQR: 0.56–0.67), *p* = 0.830). High T-stage was more common in the AUMC dataset (T3 + T4: 105 patients) compared to the NKI cohort (T3 + T4: 75 patients) (*p* = 0.014). In addition, a significant difference was found between the subsites of cancer in both cohort patients (*p* = 0.005) (tonsil: NKI = 88 (58%) vs. AUMC = 66 (42%); base of tongue: NKI = 48 (31%) vs. AUMC = 66 (42%); and posterior wall: NKI = 4 (3%) vs. AUMC = 19 (13%)). The AUMC cohort had a higher proportion of HPV-negative cases than the NKI cohort, with 50% of the NKI cohort and 69% of the AUMC cohort being HPV-negative, a statistically significant difference (*p* = 0.009).

**Table 1:** Patient demographic information of the NKI (*n* = 153 patients, The Netherlands Cancer Institute) and AUMC (*n* = 157 patients, Amsterdam UMC) cohorts

	NKI cohort	AUMC cohort	*p*-value
Age			
Median age in years [IQR]	61 [56–66]	61 [56–67]	0.830^a^
Gender (%)			
Male	96 (63)	112 (71)	0.292^b^
Smoking, *n* (%)	114 (75)	125 (80)	0.498^b^
T-stage, *n* (%)			0.014^b^
T1 + T2	78 (51)	52 (33)	
T3 + T4	75 (49)	105 (67)	
N-stage 0, *n* (%)	127 (83)	128 (82)	1^b^
Subsite of cancer, *n* (%)			0.005^b^
Tonsil	88 (58)	66 (42)	
Soft palate	13 (8)	6 (3)	
Base of tongue	48 (31)	66 (42)	
Posterior wall	4 (3)	19 (13)	
HPV, *n* (%)			0.009^b^
Negative	77 (50)	108 (69)	
Positive	76 (50)	49 (31)	

Independent Student’s *t*-test (^a^) and Chi-squared (^b^) were used to assess the differences between clinical variables

### Model performance

**Fig. 3:**
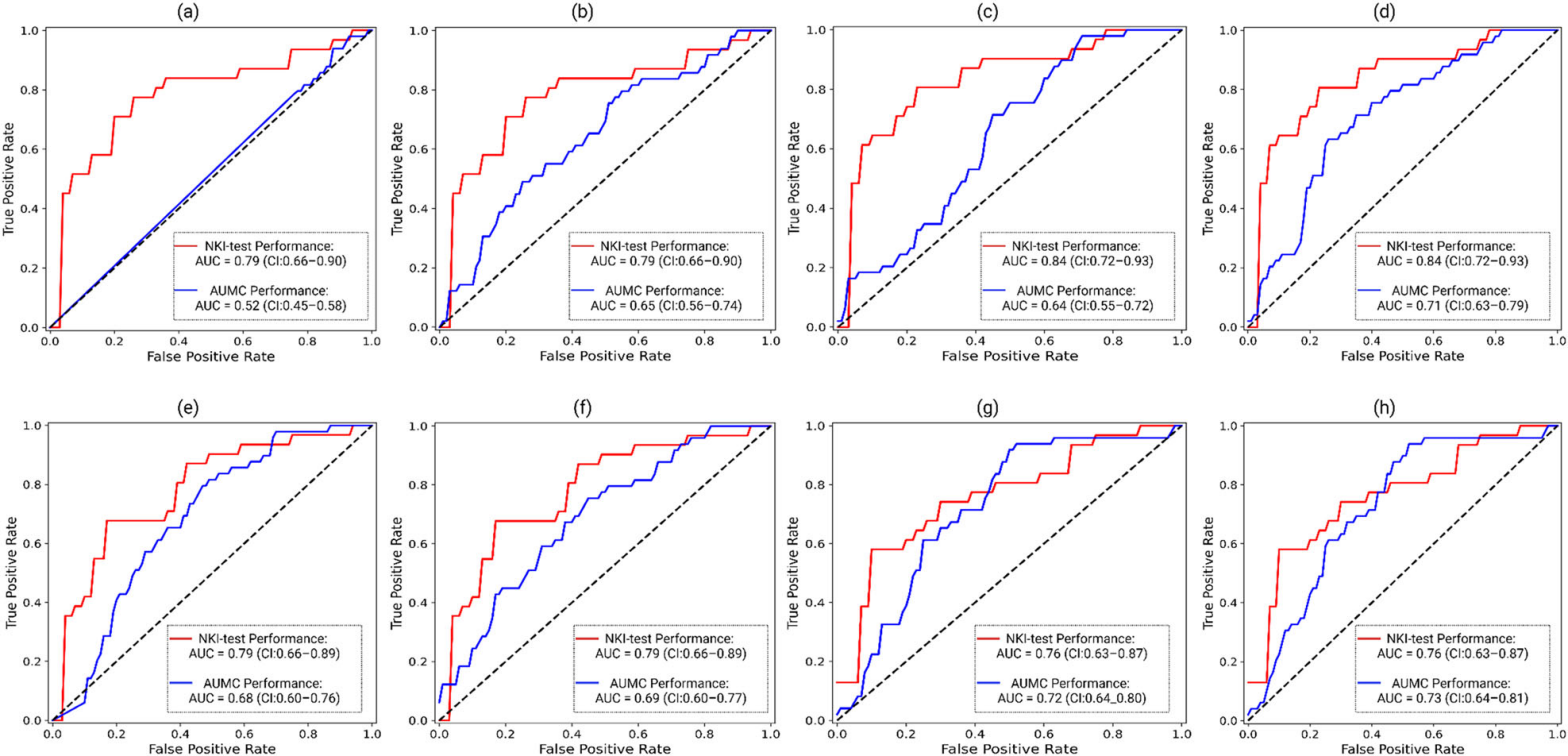
ROC-AUC curve for performance evaluation of the predictive model with diverse post-processing strategies. This figure illustrates ROC-AUC curves to assess the robustness and generalisability of the predictive model under various post-processing strategies. The evaluated post-processing steps include: **a** No post-processing, **b** Data harmonisation, **c** Stability, **d** Stability + Data harmonisation, **e** Correlation removal, **f** Correlation removal + Data harmonisation, **g** Correlation removal + Stability, and **h** Correlation removal + Data harmonisation + Stability

The results of all experiments are summarised in Fig. 3 and Table 2.

**Table 2:** Impact of post-processing steps on the generalisability of the radiogenomic predictive model

Post-processing steps on radiomic features	Identified features	Selected features by RFE	AUC		Sensitivity		Specificity		PPV		NPV		*p*-value	
			NKI	AUMC	NKI	AUMC	NKI	AUMC	NKI	AUMC	NKI	AUMC	NKI	AUMC
No post-processing	1184	53	0.79 (0.66–0.90)	0.52 (0.45–0.58)	0.82 (0.66–0.94)	1 (1.00–1.00)	0.65 (0.48–0.81)	0.02 (0.00–0.05)	0.69 (0.55–0.83)	0.32 (0.25–0.39)	0.78 (0.61–0.93)	1 (1.00–1.00)	< 0.001	0.334
Data harmonisation	1184	53	0.79 (0.66–0.90)	0.65 (0.56–0.74)	0.82 (0.66–0.94)	0.65 (0.52–0.79)	0.65 (0.48–0.81)	0.52 (0.43–0.62)	0.69 (0.55–0.83)	0.38 (0.28–0.49)	0.78 (0.61–0.93)	0.77 (0.67–0.86)	< 0.001	0.001
Correlation removal + Stability	77	3	0.76 (0.63–0.87)	0.72 (0.64–0.80)	0.75 (0.58–0.90)	0.96 (0.90–1.0)	0.70 (0.53–0.86)	0.25 (0.17–0.34)	0.72 (0.56–0.87)	0.37 (0.29–0.45)	0.74 (0.57–0.90)	0.94 (0.83–1.00)	< 0.001	< 0.001
Correlation removal + Data harmonisation + Stability	77	3	0.76 (0.63–0.87)	0.73 (0.64–0.81)	0.75 (0.58–0.90)	0.73 (0.60–0.85)	0.70 (0.53–0.86)	0.59 (0.50–0.68)	0.72 (0.56–0.87)	0.45 (0.35–0.56)	0.74 (0.57–0.90)	0.83 (0.74–0.91)	< 0.001	< 0.001

*NKI* NKI-test set, *AUMC* AUMC cohort, *RFE* recursive feature elimination

### Generalisability of HPV radiogenomic model without data post-processing

In accordance with the previously published prediction model [21] and using the radiomic features (*n* = 1184) derived from the NKI-training cohort, a model was trained to predict HPV status from baseline MRI. No post-processing steps were performed (53 out of 1184 radiomic features were selected by RFE and fed into the prediction model). This resulted in a model with reasonably good performance values in the NKI-test set (AUC = 0.79 (95% CI: 0.66–0.90, *p* < 0.001)). However, the model showed poor predictive performance when tested on the AUMC data (AUC = 0.52 (95% CI: 0.45–0.58, *p* = 0.334)) (Fig. 3a), expectantly related to the differences in scan protocols and scanners.

### Effect of data harmonisation on predictive model performance

When corrected for the impact of centre-level differences, the previously trained HPV prediction model (above and on the full set of radiomic features of the NKI-training set) showed better generalisability on the harmonised AUMC data with a predictive performance value of AUC = 0.65 (95% CI: 0.56–0.74, *p* < 0.001). As harmonisation only impacted the AUMC cohort, the predictive values remained unchanged in the NKI-test set (AUC = 0.79 (95% CI: 0.66–0.90, *p* < 0.001)) (see Fig. 3b).

### Impact of stability of radiomic features on model generalisability

To enhance the quality of the input data for the model, we explored methods to remove features from the NKI-training set that might introduce noise. We first attempted to exclude radiomic features vulnerable to differences in segmentation variability. Using this strategy, we identified 960 out of 1184 stable radiomic features with an ICC above 0.75. Subsequently, the Mann–Whitney U test was applied, resulting in the identification of 240 out of 960 features that were stable against variations in MR-image acquisition protocols. From those 240 stable radiomic features, 22 features were selected using RFE for modelling. When applying this post-processing step, the predictive model performed better with an AUC = 0.84 (95% CI: 0.72–0.93, *p* < 0.001) and an AUC = 0.64 (95% CI: 0.55–0.72, *p* = 0.002) for the NKI-test and AUMC cohorts, respectively, as shown in Fig. 3c.

Figure 3d showed that the generalisability of the trained model, which used stable radiomic features, was improved by ComBat harmonisation (AUC = 0.71 (95% CI: 0.63–0.79, *p* < 0.001)) during performance evaluation on the AUMC data.

### Correlated radiomic feature removal makes the predictive model more generalisable

From all radiomic features (1184 features), we next selected a set of uncorrelated radiomic features (259 features) to address potential issues arising from correlations among radiomic features. During feature selection, RFE identified 57 radiomic features from a total of 259 to generate the prediction model. We then assessed the model’s predictive performance using these uncorrelated features. Without harmonisation, the model’s performance reached AUC values of AUC = 0.79 (95% CI: 0.66–0.89, *p* < 0.001) in the NKI-test set and AUC = 0.68 (95% CI: 0.60–0.76, *p* < 0.001) in the AUMC cohort (Fig. 3e). After ComBat harmonisation, the predictive model achieved an AUC = 0.69 (95% CI: 0.60–0.77, *p* < 0.001) in the AUMC cohort (Fig. 3f).

To further explore performance improvement opportunities, we removed correlated radiomic features from the previously identified 240 radiomic features that exhibited stability against both observer and scan protocol variability. Within this process, 77 uncorrelated radiomic features were derived from the NKI cohort. From those 77 radiomic features, the RFE process resulted in the selection of 3 features for modelling, including original_shape_Sphericity, log-sigma-2-0-mm-3D_glcm_Idm, and wavelet-LLL_firstorder_Kurtosis. The generalisability of the predictive model improved with an AUC = 0.76 (95% CI: 0.63–0.87, *p* < 0.001) and an AUC = 0.72 (95% CI: 0.64–0.80, *p* < 0.001) for the NKI-test and AUMC cohorts, respectively (Fig. 3g). After harmonising the AUMC data, the model’s predictive performance yielded an AUC = 0.73 (95% CI: 0.64–0.81, *p* < 0.001) (Fig. 3h, and Supplementary Material S2 and S3). For further analysis, see Supplementary S4 showing the PCA of radiomic features for the NKI and AUMC cohorts under various post-processing steps.

## Discussion

This study aimed to improve the robustness and generalisability of MRI-based radiogenomic prediction models across different centres and imaging practices. A previously published MRI-based HPV status prediction model for OPSCC was used to assess the impact of post-processing steps on these model quality measures [21]. The main finding is that the predictive performance of the trained model using the NKI-training set could be successfully generalised on the AUMC cohort when following post-processing best practices, such as data harmonisation, identifying stable radiomic features, and removing highly correlated features. This study shows that these steps successfully addressed challenges posed by diverse data acquisition methods across different centres, segmentations, and scan protocols.

The model trained on the NKI-training set showed limited generalisability on the AUMC cohort, as illustrated in Fig. 3a and Table 2. Despite the high NKI-test set performance, poor model performance on the AUMC cohort may point to a lack of generalisability caused by several factors, expectantly related to the differences in scanners and image acquisition parameters across different centres, scan protocols, and segmentations.

Significant differences were observed in the imaging methodologies between the two cohorts; the NKI patient cohort utilised post-contrast 3D T1-weighted imaging with higher resolution compared to the contrast-enhanced 2D T1-weighted imaging with broader parameters used in the AUMC patient cohort (see Supplementary Material S1). These distinct imaging methodologies and hardware pose challenges to the generalisability of predictive models, as variations in imaging parameters might impact the models’ predictive performance across different cohorts [32]. In addition, the use of different scan equipment, such as 1.5-Tesla and 3.0-Tesla, introduced bias in the extracted radiomic features. This bias adversely affects the models’ generalisability [16–18]. Although we did not explicitly correct for inhomogeneities of receiver coil sensitivity, we used other established post-processing techniques to minimise variability due to technical factors. Furthermore, inherent noise in MRI scans, which refers to variations in signal intensity that are not a part of the actual signal, along with inter-observer and intra-observer variability, often leads to segmentation errors in tumour volumes. These errors are particularly challenging in head and neck tumours, complicating the accurate segmentation of these areas [19, 20]. This difficulty arises due to the large air-tissue transitions and complex anatomy in the head and neck region, which increases the possibility of segmentation errors.

We used ICC > 0.75, a standard threshold based on [26, 27], to identify radiomic features that were stable against segmentation variability (960 out of 1184 features). While the choice of this threshold involves some degree of subjectivity, it aligns with established practices for ensuring reproducibility in radiomics. Subsequently, the stable radiomic features against scan protocol variabilities were identified using the Mann–Whitney U test (240 out of 960 features). Predictive performance was increased on both the NKI-test set and the AUMC cohort when generating a model based on radiomic features that are stable across different scan protocols and inter-observer variability. This might be a result of the use of fewer radiomic features as input for predictive model generation. Fewer features reduce the complexity and can improve the robustness and generalisability of predictive models [12, 33, 34].

There was a significant gap between the performance results of the NKI-test and AUMC cohorts, highlighting the model’s struggle with generalisability. Here, we show that data harmonisation techniques are crucial to addressing this challenge in ensuring consistency and comparability across multicentre cohorts, facilitating more generalisable predictions in real-world radiogenomics models [35, 36]. The implementation of data harmonisation using the ComBat method significantly enhanced the predictive model’s performance in the AUMC cohort. This method is particularly effective in harmonising data across different centres, improving the model’s generalisability [37, 38].

Significant correlations among the radiomic features pose a notable challenge known as “multicollinearity” within the predictive model. This can lead to overfitting and unreliability, making it difficult to interpret the significance of individual features [39, 40]. In our study, removing correlated features, using the Pearson correlation coefficient method with a threshold set at > 0.9, proved to be a valuable strategy to augment the performance and generalisability of the predictive model. Notably, our results demonstrate that removing highly correlated radiomic features and identifying stable radiomic features across various scan protocols and segmentations eliminates the need for data harmonisation (see Fig. 3g, h and Table 2). This finding indicates that effective radiomic feature elimination can substitute for the harmonisation process, simplifying the post-processing workflow and enhancing the model’s generalisability across different centres.

The consideration of event per value (EPV) is essential in logistic regression model generation to ensure stability and reliability of the resulting models, as highlighted by Peeduuzi et al [41]. In radio(geno)mic studies, due to the inherent high dimensionality of radiomic features and the limited number of samples or events available [42], this is, however, often impossible and a challenge we aimed to address by assessing the impact of different post-processing steps. In our study, the initial “scenario” used the full set of 1184 radiomic features, where 53 features were selected via RFE and subsequently fed into the model. We used this configuration to serve as a reference, a baseline or “worse case” comparator, as evident from its overfitting and poor performance on the external validation set. From this base, we next applied post-processing steps to identify stable radiomic features and remove correlated ones, resulting in a reduced feature set of 77 out of 1184 radiomic features. From this subset, RFE selected 3 features, which were then used for model generation. Doing so, we achieved an EPV of > 10 (a recommended threshold). We can conclude that the model demonstrated improved stability among others by increasing EPV and addressing associated overfitting issues due to the robust post-processing steps employed.

While the outcomes are promising, it is important to note certain limitations. Our prediction model, as a proof of concept, while demonstrating the potential of MRI radiogenomics to assess a tumour genotype and generalisability across centres, could not reach the precision of biopsy-based methods for determining HPV status in OPSCC. Immunohistochemical analysis of p16 (p16^INK4a^) expression is the most widely applied method to determine HPV status [43]. Additional PCR-based HPV detection methods are strongly advised to identify p16 false positives and prevent sub-optimal prognostic information and treatment in patients [44]. Similarly, the combined analysis of p16 and p53 expression aims to improve the specificity of HPV status determination by immunohistochemistry [45]. Although the various molecular assays have their advantages and disadvantages, none is perfect, and molecular assays to detect the virus are labour-intensive, add costs to the clinical workflow, and are unavailable in some hospitals. While biopsy is the gold standard for diagnosing HPV status, future radiogenomic models could provide early insights into HPV status, which may be beneficial for risk stratification or planning treatment. While this study highlights non-invasive biomarker assessment opportunities through radiogenomics, these prediction models are neither intended (nor clinically sufficient) to replace biopsy for diagnostic precision. However, further research into radiogenomics might offer a future non-invasive tool that can help improve patient management in various clinical contexts.

Consequently, our study provides crucial insights on how post-processing steps can enhance the generalisability of radiogenomics models. These steps include data harmonisation, identifying stable radiomic features, and eliminating highly correlated features. This study also highlights the challenges that originate from varying data acquisition methods, segmentations, and scan protocols across different centres. Here, we show that these challenges can be addressed by standardising data through post-processing techniques, thereby improving the robustness and generalisability of radiomic-based prediction models.

## Supplementary information

Supplementary Information

## Abbreviations

AUC: Area under the receiver operating characteristic curve
AUMC: Amsterdam UMC
CI: Confidence interval
CT: Computed tomography
EPV: Event per value
HPV: Human papillomavirus
ICC: Intraclass correlation coefficient
IQR: Interquartile range
MRI: Magnetic resonance imaging
NKI: Netherlands Cancer Institute
NPV: Negative predictive value
OPSCC: Oropharyngeal squamous cell carcinoma
PCA: Principal component analysis
PET: Positron emission tomography
PPV: Positive predictive value
RFE: Recursive feature elimination
