# Supplementary material for: Post-processing steps improve generalisability and robustness of an MRI-based radiogenomic model for human papillomavirus status prediction in oropharyngeal cancer
Source: Eur Radiol. 2025 Jun 6;35(12):7727–37. doi: 10.1007/s00330-025-11709-8 (PMC12634727; doi:10.1007/s00330-025-11709-8)

Post-processing steps improve generalisability and robustness of an MRI-based radiogenomic model for human papillomavirus status prediction in oropharyngeal cancer

ELECTRONIC SUPPLEMENTARY MATERIAL

S1. MR image acquisition parameters utilised for the NKI and AUMC data collection.

In the NKI patient cohort, imaging was conducted in 3D, contrasting with the 2D imaging used in the AUMC cohort. The NKI cohort employed a lower slice thickness, ranging from 0.8 to 1.0 mm, compared to the AUMC cohort's slice thickness of 4.0 to 7.0 mm. In addition, pixel spacing was more variable in the NKI cohort (0.2 to 1.0 mm) than in the AUMC cohort (0.4 to 0.6 mm).

Repetition time in the NKI cohort ranged from 4.3 to 10.0 ms, wider than the 4.0 to 8.2 ms range observed in the AUMC cohort. Echo time also differed significantly between the cohorts, with the NKI cohort using a much shorter range of 1.74 to 4.6 ms compared to the 8.6 to 16.0 ms range in the AUMC cohort. Additionally, the NKI cohort used a flip angle of 10°, whereas the AUMC cohort used a flip angle of 90°. Fat suppression techniques were employed in the NKI cohort to enhance image quality, whereas no fat suppression techniques were applied in the AUMC cohort.

These differences highlight the distinct imaging protocols and methodologies between two cohorts, with the NKI cohort focusing on higher spatial resolution and detailed imaging parameters compared to the broader and more varied parameters used in the AUMC cohort. This variability could cause complicated challenges for creating a generalisable and robust HPV-predicting radiogenomic model.

S2. Comprehensive measurement of the post-processing steps on the generalisability of the predictive model in terms of various evaluation metrics. NKI; NKI-test set, AUMC; AUMC cohort, RFE; Recursive feature elimination.

Post-processing steps on radiomic features	Identified features	Selected features by RFE	AUC		Sensitivity		Specificity		PPV		NPV		p	
			NKI	AUMC	NKI	AUMC	NKI	AUMC	NKI	AUMC	NKI	AUMC	NKI	AUMC
**No post-processing**	1184	53	0.79 (0.66—0.90)	0.52 (0.45—0.58)	0.82 (0.66—0.94)	1 (1.00—1.00)	0.65 (0.48—0.81)	0.02 (0.00—0.05)	0.69 (0.55—0.83)	0.32 (0.25—0.39)	0.78 (0.61—0.93)	1 (1.00—1.00)	<0.001	0.334
**Data harmonisation**	1184	53	0.79 (0.66—0.90)	0.65 (0.56—0.74)	0.82 (0.66—0.94)	0.65 (0.52—0.79)	0.65 (0.48—0.81)	0.52 (0.43—0.62)	0.69 (0.55—0.83)	0.38 (0.28—0.49)	0.78 (0.61—0.93)	0.77 (0.67—0.86)	<0.001	0.001
**Stability**	240	22	0.84 (0.72—0.93)	0.64 (0.55—0.72)	0.81 (0.67—0.94)	0.16 (0.07—0.28)	0.71 (0.54—0.86)	0.96 (0.93—0.99)	0.74 (0.57—0.88)	0.67 (0.38—0.91)	0.79 (0.63—0.93)	0.72 (0.64—0.79)	<0.001	0.002
**Stability + Data harmonisation**	240	22	0.84 (0.72—0.93)	0.71 (0.63—0.79)	0.81 (0.67—0.94)	0.78 (0.66—0.89)	0.71 (0.54—0.86)	0.57 (0.47—0.66)	0.74 (0.57—0.88)	0.45 (0.35—0.55)	0.79 (0.63—0.93)	0.85 (0.76—0.92)	<0.001	<0.001
**Correlation removal**	259	57	0.79 (0.66—0.89)	0.68 (0.60—0.76)	0.75 (0.50—0.89)	0.30 (0.17—0.44)	0.61 (0.44—0.79)	0.82 (0.72—0.88)	0.66 (0.50—0.81)	0.43 (0.26—0.54)	0.71 (0.52—0.87)	0.72 (0.64—0.80)	<0.001	<0.001
**Correlation removal + Data harmonisation**	259	57	0.79 (0.66—0.89)	0.69 (0.60—0.77)	0.75 (0.50—0.89)	0.68 (0.54—0.80)	0.61 (0.44—0.79)	0.59 (0.50—0.69)	0.66 (0.50—0.81)	0.43 (0.33—0.54)	0.71 (0.52—0.87)	0.80 (0.71—0.89)	<0.001	<0.001
**Correlation removal + Stability**	77	3	0.76 (0.63—0.87)	0.72 (0.64—0.80)	0.75 (0.58—0.90)	0.96 (0.90—1.0)	0.70 (0.53—0.86)	0.25 (0.17—0.34	0.72 (0.56—0.87)	0.37 (0.29—0.45)	0.74 (0.57—0.90)	0.94 (0.83—1.00)	<0.001	<0.001
**Correlation removal + Data harmonisation + Stability**	77	3	0.76 (0.63—0.87)	0.73 (0.64—0.81)	0.75 (0.58—0.90)	0.73 (0.60—0.85)	0.70 (0.53—0.86)	0.59 (0.50—0.68)	0.72 (0.56—0.87)	0.45 (0.35—0.56)	0.74 (0.57—0.90)	0.83 (0.74—0.91)	<0.001	<0.001

S3. Impact of post-processing steps on the generalisabilty of predictive models. Graphs depict the effect of post-processing strategies on the generalisability of the predictive model in terms of AUC, sensitivity, specificity, PPV, and NPV metrics.
No PP; No post-processing, DH; Data harmonisation, S; Stability, CR; Correlation removal.

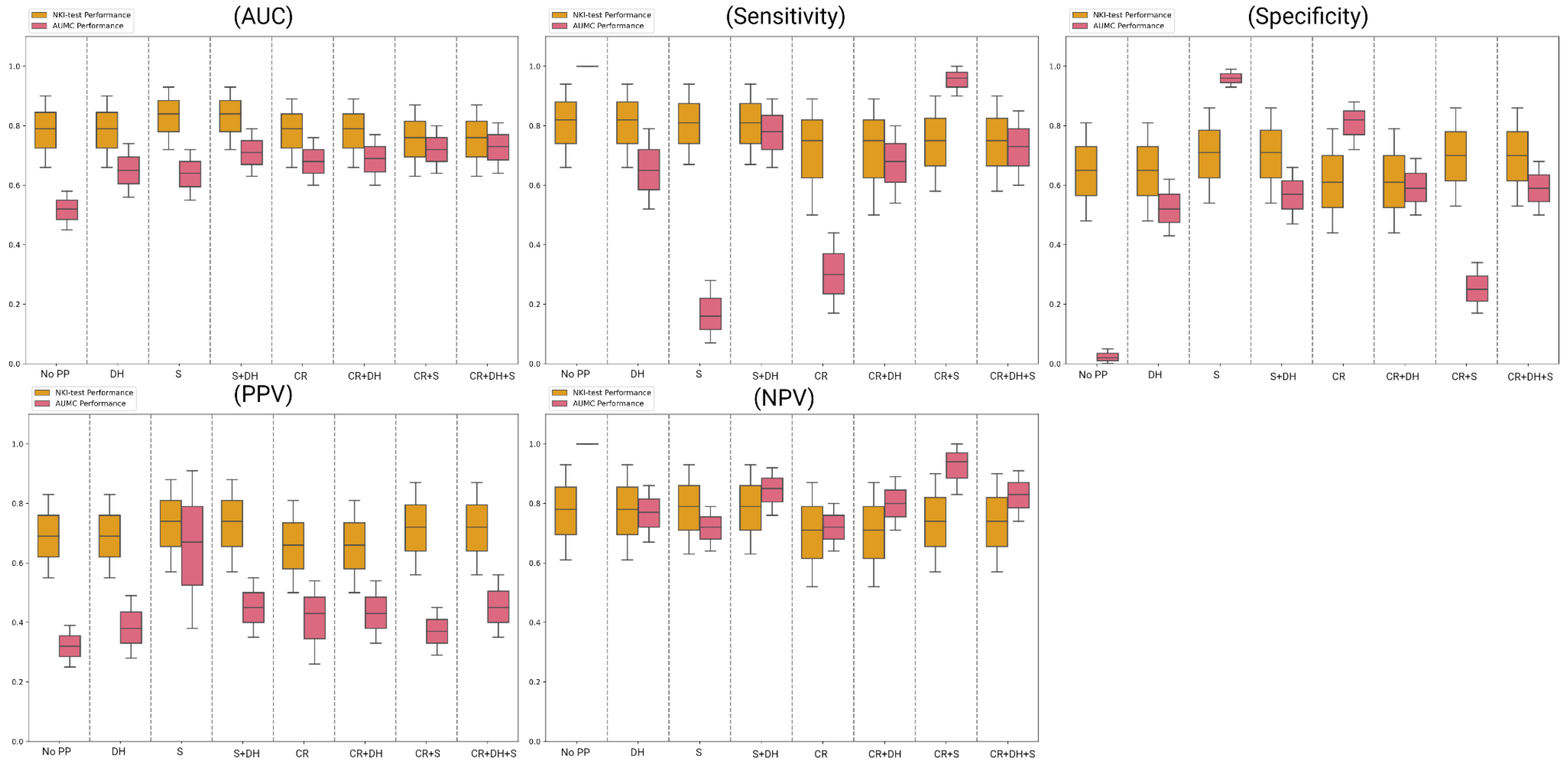

S4. Principal Component Analysis (PCA) of radiomic features for NKI and AUMC cohorts: a) PCA using All 1,184 radiomic features, b) PCA using 240 stable radiomic features, c) PCA using 77 stable and uncorrelated features without data harmonisation, and d) PCA using 77 stable and uncorrelated features with data harmonisation.
NKI; NKI cohort, AUMC; AUMC cohort.

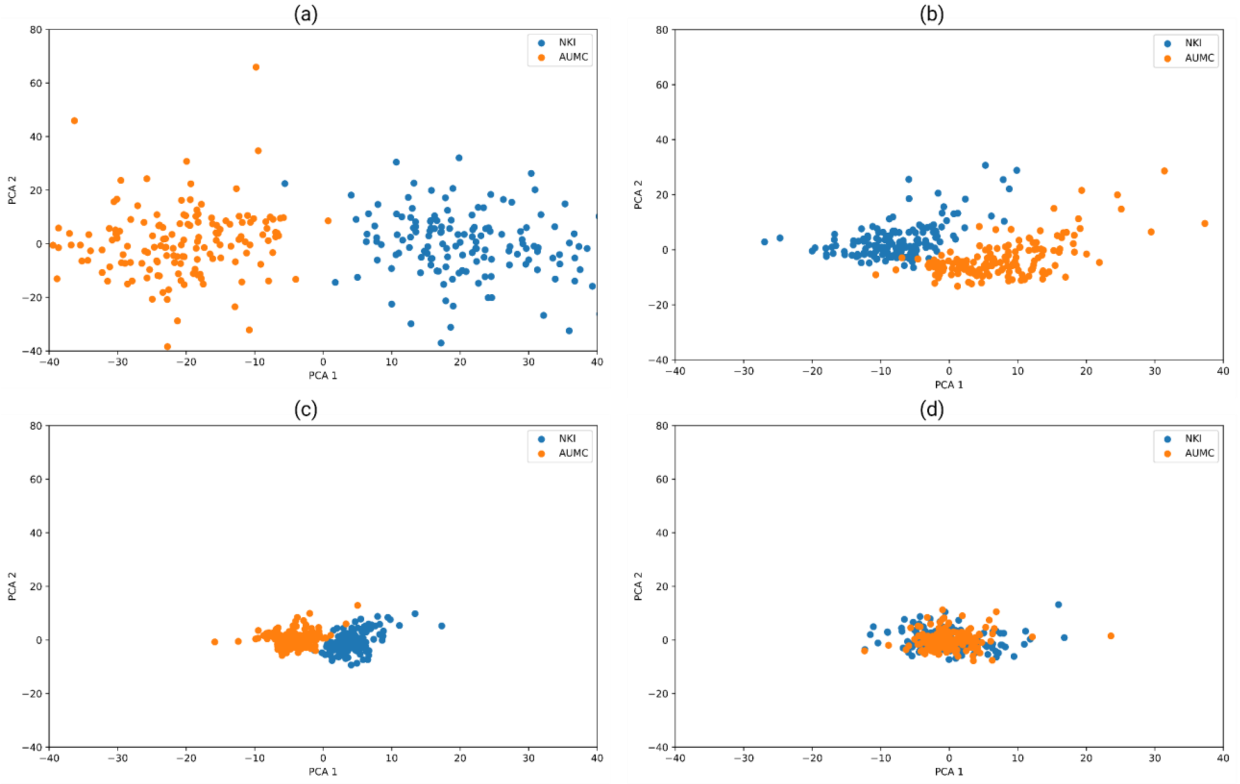

Supplement: Supplementary file 1 — Supplementary Information [file 330_2025_11709_MOESM1_ESM.pdf]
